# CO_2_ Conversion in Cu–Pd Based Disordered Network Metamaterials with Ultrasmall Mode Volumes

**DOI:** 10.1021/acs.nanolett.4c05426

**Published:** 2025-02-20

**Authors:** Jelena Wohlwend, Oliver Wipf, David Kiwic, Siro Käch, Benjamin Mächler, Georg Haberfehlner, Ralph Spolenak, Henning Galinski

**Affiliations:** † Laboratory for Nanometallurgy, Department of Materials, ETH Zurich, 8093 Zürich, Switzerland; ‡ Laboratory for Multifunctional Materials, Department of Materials, ETH Zurich, 8093 Zürich, Switzerland; ¶ Institut für Elektronenmikroskopie und Nanoanalytik, TU Graz, 8010 Graz, Austria

**Keywords:** plasmonics, disordered photonics, self-assembled metamaterials, CO_2_ conversion, local density of optical states, EELS

## Abstract

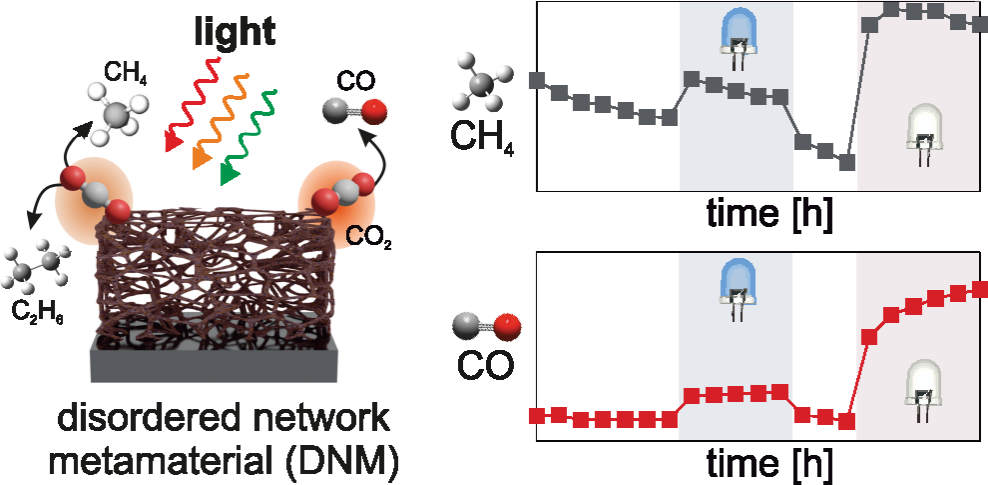

Plasmons can drive chemical reactions by directly exciting intramolecular transitions. However, strong coupling of plasmons to single molecules remains a challenge as ultrasmall mode volumes are required. In the presented work, we propose Cu–Pd plasmonic network metamaterials as scalable platforms for plasmon-assisted catalysis. Due to the absence of translational symmetry, these networks provide a unique plasmonic environment featuring a large local density of optical states and an unparalleled density of hotspots that effectively localizes light in mode volumes *V* < 8 × 10^–24^ m^3^. Catalytic performance tests during CO_2_ conversion reveal production rates of up to 4.3 × 10^2^ mmol g^–1^ h^–1^ and altered reaction selectivity under light illumination. Importantly, we show that the selectivity of the catalytic process can be tuned by modifying the network’s chemical composition, offering a versatile approach to optimize reaction pathways.

Reducing CO_2_ in our atmosphere is a necessity in order to avert the pending climate crisis caused by the continuous emission of CO_2_ through the burning of fossil fuels like coal, oil, and natural gas. For this, new technologies have to be developed to capture and store CO_2_ and possibly convert CO_2_ into new functional chemicals such as e-fuels. The conversion of CO_2_ into e-fuels is especially intriguing, as it offers a sustainable solution to close the carbon cycle.

E-fuel synthesis is commonly achieved in specifically designed antenna-reactor complexes, where nonradiative plasmonic decay is utilized to drive chemical reactions by light.^1,2^ Plasmon-assisted catalysis is a synergistic effect, and the role and interaction between the single contributions are the subject of intense scientific debates.^3−7^ Mechanisms at play include near-field enhancement, local heating, and “hot” carrier generation.^8−10^ While near-field enhancement and local heating are classical phenomena, the generation of “hot” carriers is a quantum effect. It results from Landau damping, i.e., the nonconservation of linear momentum of electrons near surfaces and regions of high field enhancement (hotspots). Most conventional plasmonic antenna-reactor designs are based on two-dimensional systems, such as metasurfaces,^11−14^ dispersed nanoparticles,^2,15,16^ and nanosheets.^17,18^ However, the low surface-to-volume ratio in such 2D systems impairs their performance. It limits both the total number of reaction sites on such surfaces and the hotspot density. This constraint is critical under realistic illumination conditions as only one plasmon polariton per nanoparticle exists at a given time and the time between excitations can be as long as microseconds.^7^ The confinement of the catalytic reactions to a plane also promotes local depletion effects of the reactants, leading to poor conversion performance.^19^ Furthermore, simple geometries such as discs or particles only allow for moderate localization of light and are inherently bandwidth limited.^20^ This further constrains the injection of “hot” carriers.^7^ The combination of large mode volumes *V* and low quality factors *Q*(14,21,22) of the resonant elements aggravates strong coupling in these two-dimensional systems.^23,24^

In this work, we propose disordered network metamaterials (DNM) as a scalable platform for plasmonic catalysis in a strong coupling regime. Unlike two-dimensional antenna-reactor systems, these self-assembled structures combine a high surface-to-volume ratio and high density of hotspots that effectively localize light in ultrasmall mode volumes *V*. Such small mode volumes *V* are expected to enhance the dipolar plasmon–molecule interaction, as its coupling strength scales with .^25^ In particular, we use Cu–Pd based plasmonic networks to show the successful design of scalable light harvesting catalysts. These DNMs enable quasi-perfect absorption of light, selectivity for specific reaction pathways, and plasmon-enhanced catalytic conversion. DNMs are fabricated by a simple two-step process: physical vapor deposition of a ternary alloy and self-assembly by chemical dealloying (Figure 1A).^26−28^

**Figure 1:**
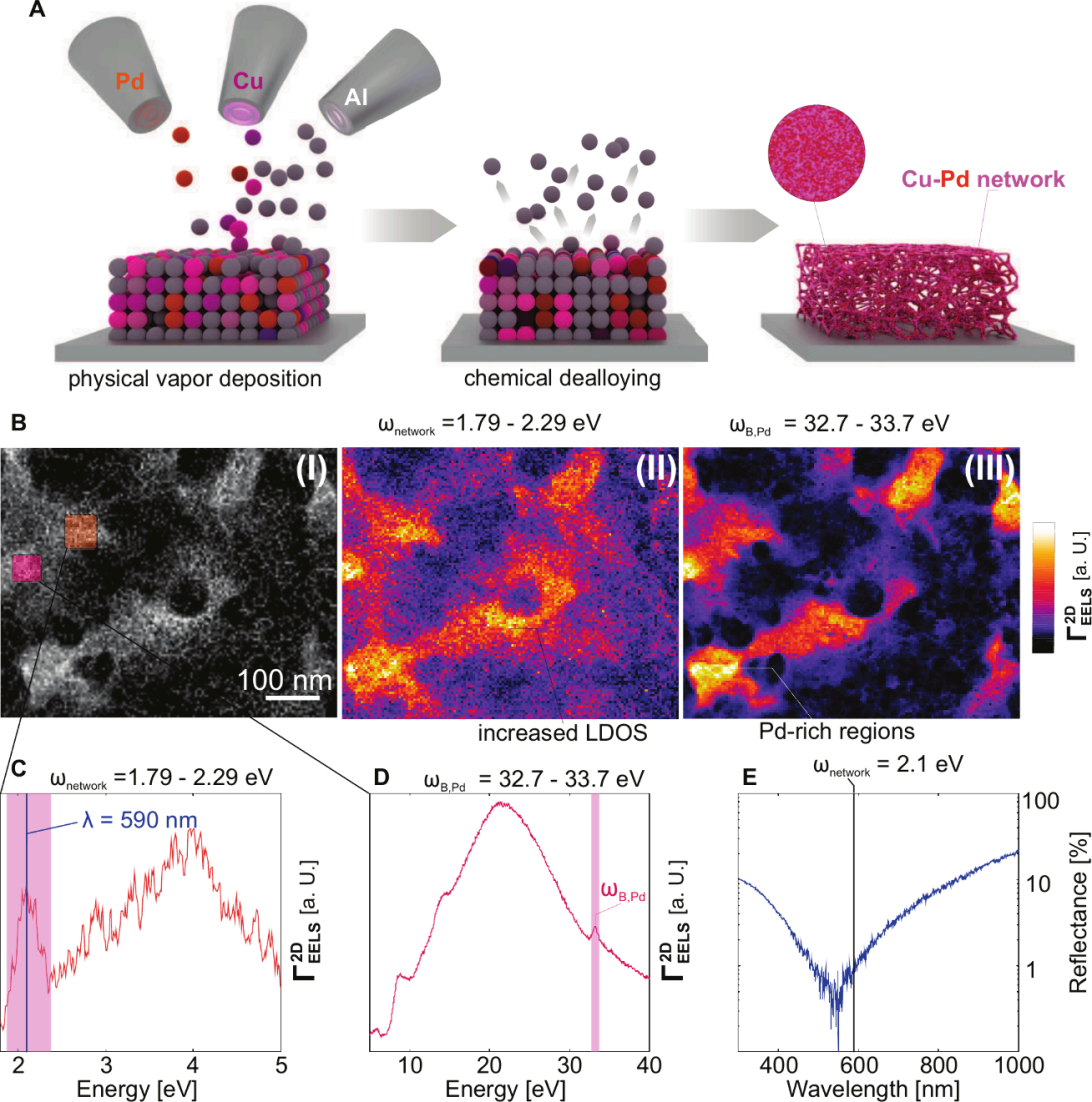
Fabrication and characterization of Cu–Pd disordered network metamaterials. (A) Fabrication of the disordered network metamaterials by a two-step process: physical vapor deposition followed by chemical dealloying. (B) (I) HAADF micrographs of a selected Cu–Pd disordered network metamaterial with a volume fraction of Cu_.75_Pd_.25_; (II) color-coded maps of global network mode (III) and bulk plasmon excitation. EEL spectra of the global network mode (C) and bulk mode (D) of the network were taken from “hot” spots. The regions are indicated with colored squares in the HAADF micrograph. (C) Localized plasmonic eigenmodes in a selected “hot” spot. The energy range corresponding to eigenmodes at visible frequencies ω_network_ is shaded in pink. (D) High energy EEL spectrum in a selected “hot” spot showing the bulk plasmon peak ω_B,Pb_ of Pd (shaded in pink). (E) Near-normal incidence reflectance spectrum of the Cu–Pd network (far field response).

The catalytic performance of the DNMs is screened using the light-assisted conversion of carbon dioxide at temperatures between 225 and 300 °C. Bimetallic Cu–Pd systems have been shown to be effective catalysts for CO_2_ hydrogenation reactions.^29,30^ The advantage of our systems lies in the capacity to modify the local chemistry and architecture of disordered networks easily during the first fabrication step. This provides a powerful approach to tailor both selectivity and absorption strength by adjusting the position of the d-band centers (see Supporting Information).^1,31^

To study the localization of light in our networks, we employ energy electron loss spectroscopy (EELS). EELS has emerged as an ideal technique to characterize plasmonic structures on the nanoscale. EELS maps not only offer insights into local plasmonic excitations but also provide detailed insights into nanoscale chemistry. For ultrathin systems, the low energy EELS signal Γ_EELS_^2D^ is directly proportional to the local density of optical states ρ (LDOS), while the high energy signal correlates with the electron density^32−36^ (Figure 1C,D). The LDOS describes the electromagnetic environment of a dipolar emitter and critically impacts the light–matter interaction, e.g., intermolecular transitions in a plasmonic cavity.^37^

In Figure 1B (I) a high angle annular dark field (HAADF) micrograph with corresponding EEL maps of a Cu–Pd network, with a volume of fraction of Cu_.75_Pd_.25_, is shown. The HAADF micrograph confirms the formation of a nanosized network.

To visualize the distribution of the plasmonic modes, we integrate the EELS signal within a spectral window from 1.79 to 2.29 eV (Figure 1B (II)). We observe large fluctuations in the LDOS modulated within the network. Such fluctuations confirm both an increased contribution of localized plasmonic eigenmodes and their ability to localize energy at nanometric scales in so-called “hotspots“.^37^ Measurements of the spatial extension of these modes reveal typical mode volumes of 20 × 20 × 20 nm^3^. By analyzing the bulk plasmon peak (Figure 1B (III)) we can link these fluctuations in LDOS to modulations in the Pd content.

Within the hotspots, we observe a bimodal spectrum of plasmonic modes. One set of modes is centered at 2.1 eV, corresponding to visible wavelengths (≈590 nm), and the other set of modes centered at 3.95 eV corresponding to ultraviolet wavelength (≈313 nm)(Figure 1C). Interestingly, the plasmonic modes can also be accessed by far-field techniques, such as reflectance measurements. The far-field optical response, i.e., the reflectance spectrum in Figure 1E, corresponds well to the plasmonic eigenmodes in the visible regime in Figure 1C. We observe quasi-perfect absorption over a broad range of wavelength. The slight deviation most probably stems from the difference in excitation (photons or electrons).

In regions of high LDOS and small mode volumes *V*, i.e., high near-field enhancement, the potential energy surface of catalytic reaction is prone to change. Two specific scenarios are of interest, namely, the reduction of the activation energy *E*_a,*j*_ and the change in selectivity if different products are formed. Especially the change in selectivity implicates nonthermal effects as the total power *Q* dissipated in the metamaterial due to plasmon-induced heating is not reaction specific and proportional to the local optical losses *Q* ∼ Im(ϵ).^38^ It is of note that a high LDOS and high optical loss should generally enhance the reaction rate as the temperature is locally increased.

To study the impact of the plasmonic environment of our DNMs on their catalytic performance, we carried out CO_2_ conversion experiments in a custom build reactor (Figure 2A). Thereby, the sample is placed into a reaction chamber which is sealed by a quartz glass window and heated up (between 225 and 300 °C) while the chamber is flushed with CO_2_ and H_2_. The gas mixture escaping the reactor is directed into a gas chromatograph, which identifies the reaction products based on their distinct retention times.

**Figure 2:**
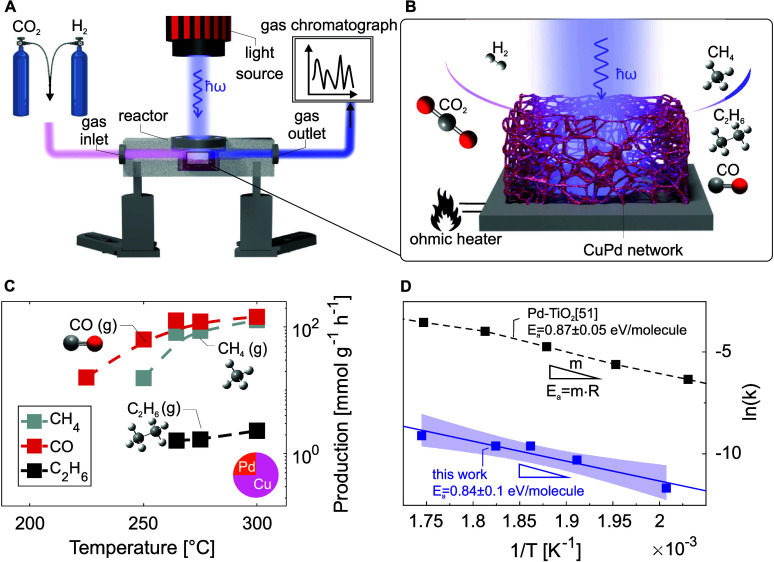
Temperature-dependent catalytic conversion of CO_**2**_. (A, B) Schematic illustration of the custom-built reactor, reactants and products, with gas inlet on the left and gas outlet on the right leading to the gas chromatograph. The chamber is sealed by a quartz window and a high-temperature O-ring, enabling illumination of the sample with a LED light source (white or UV = 365 nm). The reactor can be heated by an ohmic heater (*T*_max_ = 300 °C). (C) Temperature dependent production of CO, CH_4_, and C_2_H_6_ without optical illumination with a 75% Cu 25% Pd network. (D) Arrhenius plot of the catalytic conversion of CO_2_ to CO over Cu_.75_Pd_.25_. The temperature dependent reaction constant *k* for the RWGS reaction over Pd-TiO_2_ is plotted for comparison. The activation energy *E*_a_ = *m*·*R* = 0.84(10) eV/molecule for the conversion of CO_2_ to CO is estimated by linear regression (blue line) with a 95% confidence interval (blue band).

During the catalytic conversion of CO_2_, three main types of products are expected: CO, methanol, and hydrocarbons^49^ (Figure 2B). CO can be produced via a reverse water-gas shift (RWGS) reaction, an endothermic process in which CO_2_ is hydrogenated to produce CO, H_2_ and water, as shown in eq 1.
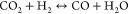
1The resulting CO can be further utilized to synthesize methanol or as feedstock in the Fischer–Tropsch reaction to produce e-fuels.^50^ In addition to CO various hydrocarbons can also be synthesized. For example, CH_4_ (methane) is desirable as an e-fuel or as a feedstock for producing synthetic natural gas (SNG). Longer-chain hydrocarbons, such as C_2_H_6_ (ethane), are valuable building blocks for further chemical synthesis or as components in fuels. Beyond the reaction products, the selectivity of the catalyst is also of great importance, as it allows us to choose a product. If a catalyst provides high selectivity, then tedious purification processes can be avoided.

In the first experiments without illumination, the temperature was gradually increased from 150 to 300 °C in steps of 25 °C (Figure 2C). At an onset temperature of 225 °C, the CO production starts, whereas the CH_4_ production only starts at slightly higher temperatures of 250 °C. The production of ethane (C_2_H_6_) starts at 265 °C. At 300 °C the network converts CO_2_ into CO, CH_4_, and C_2_H_6_ with a selectivity of 60% for CO, 39% for CH_4_, and 1% for C_2_H_6_. The activation energy can be derived from the variation of yield with temperature (for details on the calculation, see Supporting Information). The calculated activation energy for the reduction of CO_2_ to CO is *E*_a_ = 0.84(13) eV/molecule (Figure 2C) and comparable with findings by Frei et al.^51^ on the activation energy of the RWGS reaction over a Pd-TiO_2_ catalyst.

In a second set of experiments, we evaluate the influence of light on the catalytic conversion of CO_2_ at a constant temperature of 300 °C by illuminating the metamaterial with two different light sources: a 200 mW/cm^2^ LED at 365 nm and 400 mW/cm^2^ white LED (400–800) nm (Figure 3A). In the case of a Cu_.75_Pd_.25_ DNM, the production of all three reaction products is significantly enhanced by light. Specifically, the maximal enhancement is 1.3 for CH_4_, 1.7 for CO, and 1.4 for C_2_H_6_ with white light illumination (Figure 3A). Notably, the ethane production exhibits a distinct increase in selectivity between UV and white light. While UV illumination yields no significant increase in production, the presence of white light enhances ethane production by a factor of 1.4. This finding is important as it suggests a frequency dependence of the process, likely correlating with the plasmonic response measured by EELS (resonance at 2.1 eV = 590 nm).

**Figure 3:**
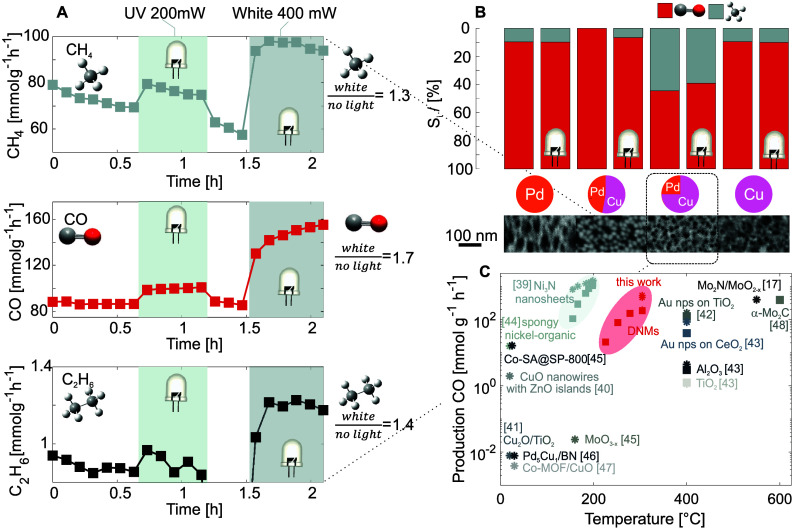
“Light-assisted” CO_2_ conversion. (A) “Light-assisted” CO_2_ conversion at constant temperature (*T*_max_ = 300 °C) as a function of time. The conversion enhancement with white LED illumination (vs no light) is depicted on the right. Especially, the C_2_H_6_ production shows a clear selectivity increase between UV irradiation (200 mW) and white light irradiation (400 mW). (B) Composition dependent selectivity *S*_*i*_ for disordered network metamaterials with different Pd/Cu content and the corresponding scanning electron micrographs showing the corresponding nanostructures of the networks. The selectivity data with illumination refer to illumination with white light. Tabulated values of the selectivity are given in the Supporting Information. (C) Comparison of the temperature dependent CO production of Cu–Pd networks with state-of-the-art catalyst from literature.^17,39−48^ Values denoted by a □ refer to production of CO without illumination, while values denoted by a ★ indicate catalysis in the presence of light.

Aside the enhancement of the catalytic activity under illumination, we observe a linear decay in CH_4_ production as a function of time under dark condition (Figure 3A) whereas the CO and C_2_H_6_ production rate remain constant. This decay, also present in the other Pd-containing networks (see Supporting Information), most likely stems from a hydrogen-induced structural instability^52^ that causes a slight coarsening of the network architectures, as shown by FIB tomography (see Supporting Information).

By tuning the network composition, both the product selectivity and activity of the DNMs can be adjusted (Figure 3B). Scanning electron microscopy images (Figure 3B) exhibit nanometric disordered network topologies that are characteristic of chemical dealloying.^53^ A detailed description of the network architectures is given in the Supporting Information. In this context, Cu_.75_Pd_.25_ bimetallic networks achieve a considerable specific surface area SSA (10 m^2^/g) as well as very small strut length *l*_s_ = 22 nm and pore intercept length *L*_m_ = 11 nm (Table 1). Especially, the small pore intercept length should favor the confinement of light to ultrasmall mode volumes.

**Table 1:** Structural Analysis of DNMs^a^

	SSA (m^2^/g)	*l*_s_ (nm)	ϕ (%)	*L*_m_ (nm)
Cu	5.5 ± 0.5	30 ± 10	37.8 ± 1.5	18 ± 3
Cu_.75_Pd_.25_	11.0 ± 1.0	22 ± 6	26.3 ± 2.6	11 ± 2
Cu_.57_Pd_.43_	6.4 ± 1.4	23 ± 4	30.3 ± 4.2	20 ± 2
Pd	11.8 ± 3.0	42 ± 12	26.2 ± 3.7	20 ± 5

a Measured initial specific surface area SSA, mean strut length *l*_s_, void fraction ϕ, and mean pore intercept length *L*_m_ of Cu–Pd plasmonic networks.

Notably, the bimetallic networks change both their selectivity and activity (Figure 3B, Supporting Information) under low-intensity illumination (≈ 0.5 W·cm^–2^). While in Cu_.75_Pd_.25_ the production of CO is enhanced, in Cu_.57_Pd_.43_ an uptick in CH_4_ production is observed. Such alteration of the reaction pathway can be linked to changes in chemistry, impacting on the binding and dissociation energy of the catalyst.^54^ Still, this change in the selectivity suggests a plasmonic contribution to the catalytic conversion of CO_2_. Yet, it remains to be determined if this plasmon induced enhancement result from field enhancement or “hot” carrier generation.

In pure Pd and Cu networks, no change in selectivity upon illumination is observed. Although the activity in pure Cu increases under light conditions, the production of Pd is not enhanced when illuminated (see also Supporting Information).

In order to compare the measured catalytic performance of our plasmonic networks with literature, we calculate the weight *g*_cat_ of the DNMs using focused-ion-beam tomography (see Supporting Information).

Cu–Pd disordered network metamaterials exhibit catalytic performances ranging from 1.46 to 98.15 mmol·g_cat_^–1^ h^–1^ for the production of CH_4_ and from 16.49 to 430.53 mmol·g_cat_^–1^ h^–1^ for the production of CO across the network compositions. Notably, the catalytic performance of our plasmonic Cu–Pd based networks is on par with state-of-the-art high performing catalysts, such as Mo_2_N/MoO_2–*x*_^17^ or Ni_3_N nanosheets^39^ (see Figure 3C). We attribute this high performance of our DNMs to the ultrasmall mode volumes which are a direct result of the self-assembly process that determines the architecture and chemical modulation of the plasmonic networks. Furthermore, the DNMs extend efficient plasmon-assisted catalysis to an intermediate operation temperature window (225–300 °C).

In conclusion, we demonstrate conversion of CO_2_ into CO, CH_4_, and C_2_H_6_ with Cu–Pd disordered network metamaterials at elevated temperatures. The reaction selectivity and catalytic yield can be tailored through chemical engineering of the bimetallic Cu–Pd system. Probing the plasmonic environment on the nanoscale, we show that regions with a high local density of states coincide with a high Pd content. In these regions light is localized in ultrasmall volumes. This combination of high local density of states and small mode volumes makes our systems ideal for plasmon assisted catalysis. Evidence of this is given by the changes in the yield and selectivity of reaction products under light illumination. It is anticipated that the selectivity and therefore also the total production rate can further be enhanced by fine-tuning the chemistry of the plasmonic networks or substituting Pd with other catalytic metals such at Ru. Furthermore, measuring the quantum efficiency, how effectively a photocatalyst converts an absorbed photon into a product, could provide further insights into the underlying mechanisms.

## Materials and Methods

### Catalyst Fabrication

The thin film catalysts were fabricated in two steps. First, Al–Cu–Pd thin films (nominal 300 nm) were cosputtered onto SiO_2_/Si wafers (381 ± 25 μm). The chemical composition of the as-deposited film is controlled by adjusting the sputtering rates of the different sources and controlling their specific power. Second, the films were chemically dealloyed in a 1 M NaOH aqueous solution, to form a disordered open-porous nanonetwork.^27,28,36^

### Materials Characterization

Focused ion beam cross sections of the thin film catalyst networks were acquired using a NVision40 focused ion beam scanning electron microscope (FIB-SEM), operated at 3 kV and with a focused Ga^+^ liquid metal ion source at 30 kV acceleration voltage. From the cross section, the surface area of the nanonetwork and the weight were calculated by importing the crosssection into the open source software Blender and creating a 3D model (Supporting Information). The network composition was determined using a liquid nitrogen cooled EDX system (EDAX). The reflectivity of the samples was measured using a deuterium/halogen lamp (Ocean Optics DH-2000-BAL) and a UV–vis–NIR (188–1033 nm wavelength) spectrometer (Ocean Optics USB2000+XR). As a calibration standard, an aluminum mirror was used.

EEL spectra of the thin film catalyst were obtained by a monochromated FEI Titan 60-300 with an imaging filter (Gatan GIF Quantum) operated in scanning mode at 300 kV. The spectra were acquired with a dispersion of 25 and 10 meV per pixel. All spectra were treated with the HQ Dark Correction plugin. Additionally, postprocessing of the EELS data included the alignment of the spectra with respect to the position of the zero-loss peak (ZLP), normalization of the maximum intensity of the ZLPs in each pixel and removal of the ZLP by fitting a premeasured ZLP using the Matlab Spectrum image analysis tool and DigitalMicrograph (Gatan).^36^

### Photocatalytic Activity Measurements

The photocatalytic conversion of CO_2_ was carried out in the gas phase in a gas flow control system (Alicat Scientific), a custom-built heatable (max 300 °C) photoreactor (Figure 2A) equipped with different light sources (Thorlabs), and a gas chromatograph (GC) (Micro GC Fusion, Inficon) for gas analysis. Thereby, the GC is equipped with three modules (Rt-Molsieve 5A with Rt-Q-Bond Backflush, Rt-QBond, and Rt-U-Bond) and thermal conductivity detectors. Representative GC spectra and light-assisted conversion experiments on the Cu, Pd, and Cu_.57_Pd_.43_ DNMs are given in the Supporting Information.

For catalytic testing, the thin film samples were loaded into the reactor chamber. The chamber was then purged with N_2_. After the purging, a gas flux (9:1 H_2_:CO_2_) was introduced. The (photo)thermal catalytic data were then recorded at different temperatures and for different network compositions.

## Supporting Information Available

Additional experimental details, materials, and methods, including analysis of network architecture, surface area and weight calculation of Cu–Pd disordered network metamaterials, calculation of activation energy for CO_2_ reduction, projected DOS of plasmonic and catalytic metals, and additional catalytic conversion data (PDF)

## References

[ref1] Sytwu K.; Vadai M.; Dionne J. A. Bimetallic nanostructures: combining plasmonic and catalytic metals for photocatalysis. Advances in Physics: X 2019, 4, 1619480 10.1080/23746149.2019.1619480.

[ref2] Zhang C.; Zhao H.; Zhou L.; Schlather A. E.; Dong L.; McClain M. J.; Swearer D. F.; Nordlander P.; Halas N. J. Al–Pd Nanodisk Heterodimers as Antenna–Reactor Photocatalysts. Nano Lett. 2016, 16, 6677–6682. 10.1021/acs.nanolett.6b03582. 27676189

[ref3] Verma R.; Sharma G.; Polshettiwar V. The paradox of thermal vs. non-thermal effects in plasmonic photocatalysis. Nat. Commun. 2024, 15, 7974 10.1038/s41467-024-51916-3. 39266509 PMC11393361

[ref4] Kazuma E.; Jung J.; Ueba H.; Trenary M.; Kim Y. Real-space and real-time observation of a plasmon-induced chemical reaction of a single molecule. Science 2018, 360, 521–526. 10.1126/science.aao0872. 29724952

[ref5] Habteyes T. G. Anions as Intermediates in Plasmon Enhanced Photocatalytic Reactions. J. Phys. Chem. C 2020, 124, 26554–26564. 10.1021/acs.jpcc.0c08831.

[ref6] Li K.; Hogan N. J.; Kale M. J.; Halas N. J.; Nordlander P.; Christopher P. Balancing Near-Field Enhancement, Absorption, and Scattering for Effective Antenna–Reactor Plasmonic Photocatalysis. Nano Lett. 2017, 17, 3710–3717. 10.1021/acs.nanolett.7b00992. 28481115

[ref7] Khurgin J. B. Fundamental limits of hot carrier injection from metal in nanoplasmonics. Nanophotonics 2020, 9, 453–471. 10.1515/nanoph-2019-0396.

[ref8] Mascaretti L.; Naldoni A. Hot electron and thermal effects in plasmonic photocatalysis. J. Appl. Phys. 2020, 128, 041101 10.1063/5.0013945.

[ref9] Fusco Z.; Beck F. J. Advances in fundamentals and application of plasmon-assisted CO2 photoreduction. Nanophotonics 2024, 13, 387–417. 10.1515/nanoph-2023-0793. 39635649 PMC11501834

[ref10] Besteiro L. V.; Kong X.-T.; Wang Z.; Hartland G.; Govorov A. Understanding Hot-Electron Generation and Plasmon Relaxation in Metal Nanocrystals: Quantum and Classical Mechanisms. ACS Photonics 2017, 4, 2759–2781. 10.1021/acsphotonics.7b00751.

[ref11] Dongare P. D.; Zhao Y.; Renard D.; Yang J.; Neumann O.; Metz J.; Yuan L.; Alabastri A.; Nordlander P.; Halas N. J. A 3D plasmonic antenna-reactor for nanoscale thermal hotspots and gradients. ACS Nano 2021, 15, 8761–8769. 10.1021/acsnano.1c01046. 33900744

[ref12] Sytwu K.; Vadai M.; Hayee F.; Angell D.; Dai A.; Dixon J.; Dionne J. Driving energetically unfavorable dehydrogenation dynamics with plasmonics. Science 2021, 371, 280–283. 10.1126/science.abd2847. 33446555

[ref13] Mascaretti L.; Schirato A.; Fornasiero P.; Boltasseva A.; Shalaev V. M.; Alabastri A.; Naldoni A. Challenges and prospects of plasmonic metasurfaces for photothermal catalysis. Nanophotonics 2022, 11, 3035–3056. 10.1515/nanoph-2022-0073. 39634672 PMC11501173

[ref14] Yuan L.; Zhao Y.; Toma A.; Aglieri V.; Gerislioglu B.; Yuan Y.; Lou M.; Ogundare A.; Alabastri A.; Nordlander P.; et al. A Quasi-Bound States in the Continuum Dielectric Metasurface-Based Antenna-Reactor Photocatalyst. Nano Lett. 2024, 24, 172–179. 10.1021/acs.nanolett.3c03585. 38156648

[ref15] Herran M.; Juergensen S.; Kessens M.; Hoeing D.; Köppen A.; Sousa-Castillo A.; Parak W. J.; Lange H.; Reich S.; Schulz F.; et al. Plasmonic bimetallic two-dimensional supercrystals for H_2_ generation. Nature Catalysis 2023, 6, 1205–1214. 10.1038/s41929-023-01053-9.

[ref16] Kang Y.; João S. M.; Lin R.; Liu K.; Zhu L.; Fu J.; Cheong W.-C.; Lee S.; Frank K.; Nickel B.; et al. Effect of crystal facets in plasmonic catalysis. Nat. Commun. 2024, 15, 3923 10.1038/s41467-024-47994-y. 38724494 PMC11519563

[ref17] Wan X.; Li Y.; Chen Y.; Ma J.; Liu Y.-A.; Zhao E.-D.; Gu Y.; Zhao Y.; Cui Y.; Li R.; Liu D.; Long R.; Liew K.; Xiong Y. A nonmetallic plasmonic catalyst for photothermal CO_2_ flow conversion with high activity, selectivity and durability. Nat. Commun. 2024, 15, 1273 10.1038/s41467-024-45516-4. 38341405 PMC10858932

[ref18] Wang M.; Jia J.; Meng Z.; Xia J.; Hu X.; Xue F.; Peng H.; Meng X.; Yi J.; Chen X.; et al. Plasmonic Pd-Sb nanosheets for photothermal CH_4_ conversion to HCHO and therapy. Sci. Adv. 2024, 10, eado9664 10.1126/sciadv.ado9664. 39231231 PMC11373601

[ref19] Soto A. M.; Lake J. R.; Varanasi K. K. Transient Effects Caused by Gas Depletion during Carbon Dioxide Electroreduction. Langmuir 2022, 38, 1020–1033. 10.1021/acs.langmuir.1c02540. 35014259

[ref20] Chen W.-J.; Hou B.; Zhang Z.-Q.; Pendry J.; Chan C. Metamaterials with index ellipsoids at arbitrary k-points. Nat. Commun. 2018, 9, 2086 10.1038/s41467-018-04490-4. 29802280 PMC5970243

[ref21] Naya S.-i.; Tada H. Au-Ag alloy nanoparticle-incorporated AgBr plasmonic photocatalyst. Sci. Rep. 2020, 10, 19972 10.1038/s41598-020-77062-6. 33203927 PMC7673129

[ref22] Bi X.; Li Z.; Zhang C.; You Q.; Li Y.; Wang Y.; Wang P. Strong coupling of Ag@ Au hollow nanocube/J-aggregate heterostructures by absorption spectra. J. Phys. Chem. C 2022, 126, 10566–10573. 10.1021/acs.jpcc.2c02516.

[ref23] Bitton O.; Haran G. Plasmonic cavities and individual quantum emitters in the strong coupling limit. Accounts of chemical research 2022, 55, 1659–1668. 10.1021/acs.accounts.2c00028. 35649040 PMC9219108

[ref24] Lee Y.-M.; Kim S.-E.; Park J.-E. Strong coupling in plasmonic metal nanoparticles. Nano Convergence 2023, 10, 34 10.1186/s40580-023-00383-5. 37470924 PMC10359241

[ref25] Yoo D.; de León-Pérez F.; Pelton M.; Lee I.-H.; Mohr D.; Raschke M.; Caldwell J.; Martín-Moreno L.; Oh S.-H. Ultrastrong plasmon–phonon coupling via epsilon-near-zero nanocavities. Nat. Photonics 2021, 15, 125 10.1038/s41566-020-00731-5.

[ref26] Galinski H.; Fratalocchi A.; Döbeli M.; Capasso F. Light Manipulation in Metallic Nanowire Networks with Functional Connectivity. Adv. Opt. Mater. 2017, 5, 1600580 10.1002/adom.201600580.

[ref27] Wohlwend J.; Sologubenko A. S.; Döbeli M.; Galinski H.; Spolenak R. Chemical Engineering of Cu–Sn Disordered Network Metamaterials. Nano Lett. 2022, 22, 853–859. 10.1021/acs.nanolett.1c03545. 34738817

[ref28] Wohlwend J.; Hilti A.; Polinari C.; Spolenak R.; Galinski H. Hybrid Resonant Metasurfaces with Configurable Structural Colors. Adv. Opt. Mater. 2024, 12, 2401501 10.1002/adom.202401501.

[ref29] Bai S.; Shao Q.; Wang P.; Dai Q.; Wang X.; Huang X. Highly Active and Selective Hydrogenation of CO_2_ to Ethanol by Ordered Pd—Cu Nanoparticles. J. Am. Chem. Soc. 2017, 139, 6827–6830. 10.1021/jacs.7b03101. 28485583

[ref30] Jiang X.; Koizumi N.; Guo X.; Song C. Bimetallic Pd–Cu catalysts for selective CO_2_ hydrogenation to methanol. Applied Catalysis B: Environmental 2015, 170–171, 173–185. 10.1016/j.apcatb.2015.01.010.

[ref31] Yin Z.; Gao D.; Yao S.; Zhao B.; Cai F.; Lin L.; Tang P.; Zhai P.; Wang G.; Ma D.; Bao X. Highly selective palladium-copper bimetallic electrocatalysts for the electrochemical reduction of CO_2_ to CO. Nano Energy 2016, 27, 35–43. 10.1016/j.nanoen.2016.06.035.

[ref32] Kociak M.; Stéphan O. Mapping plasmons at the nanometer scale in an electron microscope. Chem. Soc. Rev. 2014, 43, 3865–3883. 10.1039/c3cs60478k. 24604161

[ref33] Hörl A.; Haberfehlner G.; Trügler A.; Schmidt F.-P.; Hohenester U.; Kothleitner G. Tomographic imaging of the photonic environment of plasmonic nanoparticles. Nat. Commun. 2017, 8, 37 10.1038/s41467-017-00051-3. 28652567 PMC5484695

[ref34] Flauraud V.; Bernasconi G. D.; Butet J.; Alexander D. T. L.; Martin O. J. F.; Brugger J. Mode Coupling in Plasmonic Heterodimers Probed with Electron Energy Loss Spectroscopy. ACS Nano 2017, 11, 3485–3495. 10.1021/acsnano.6b08589. 28290663

[ref35] Simon T.; Li X.; Martin J.; Khlopin D.; Stéphan O.; Kociak M.; Gérard D. Aluminum Cayley trees as scalable, broadband, multiresonant optical antennas. Proc. Natl. Acad. Sci. U. S. A. 2022, 119, e2116833119 10.1073/pnas.2116833119. 35046038 PMC8794834

[ref36] Wohlwend J.; Haberfehlner G.; Galinski H. Strong Coupling in Two-Phase Metamaterials Fabricated by Sequential Self-Assembly. Advanced. Opt. Mater. 2023, 11, 2300568 10.1002/adom.202300568.

[ref37] Krachmalnicoff V.; Castanié E.; De Wilde Y.; Carminati R. Fluctuations of the Local Density of States Probe Localized Surface Plasmons on Disordered Metal Films. Phys. Rev. Lett. 2010, 105, 183901 10.1103/PhysRevLett.105.183901. 21231105

[ref38] Baffou G.; Quidant R.; Girard C. Heat generation in plasmonic nanostructures: Influence of morphology. Appl. Phys. Lett. 2009, 94, 153109 10.1063/1.3116645.

[ref39] Singh S.; Verma R.; Kaul N.; Sa J.; Punjal A.; Prabhu S.; Polshettiwar V. Surface plasmon-enhanced photo-driven CO_2_ hydrogenation by hydroxy-terminated nickel nitride nanosheets. Nat. Commun. 2023, 14, 2551 10.1038/s41467-023-38235-9. 37137916 PMC10156734

[ref40] Wang W.-N.; Wu F.; Myung Y.; Niedzwiedzki D. M.; Im H. S.; Park J.; Banerjee P.; Biswas P. Surface Engineered CuO Nanowires with ZnO Islands for CO_2_ Photoreduction. ACS Appl. Mater. Interfaces 2015, 7, 5685–5692. 10.1021/am508590j. 25723846

[ref41] Jeong S.; Kim G.-M.; Kang G.-S.; Kim C.; Lee H.; Kim W.-J.; Lee Y. K.; Lee S.; Kim H.; Lim H. K.; Lee D. C. Selectivity Modulated by Surface Ligands on Cu_2_O/TiO_2_ Catalysts for Gas-Phase Photocatalytic Reduction of Carbon Dioxide. J. Phys. Chem. C 2019, 123, 29184–29191. 10.1021/acs.jpcc.9b05780.

[ref42] Martínez Molina P.; Bossers K. W.; Wienk J. D.; Rohlfs J.; Meulendijks N.; Verheijen M. A.; Buskens P.; Sastre F. Sunlight Powered Continuous Flow Reverse Water Gas Shift Process Using a Plasmonic Au/TiO_2_ Nanocatalyst. Chem.—Asian J. 2023, 18, e202300405 10.1002/asia.202300405. 37249160

[ref43] Upadhye A. A.; Ro I.; Zeng X.; Kim H. J.; Tejedor I.; Anderson M. A.; Dumesic J. A.; Huber G. W. Plasmon-enhanced reverse water gas shift reaction over oxide supported Au catalysts. Catal. Sci. Technol. 2015, 5, 2590–2601. 10.1039/C4CY01183J.

[ref44] Cui Y.; Labidi A.; Liang X.; Huang X.; Wang J.; Li X.; Dong Q.; Zhang X.; Othman S. I.; Allam A. A.; Bahnemann D. W.; Wang C. Pivotal Impact Factors in Photocatalytic Reduction of CO_2_ to Value-Added C1 and C2 Products. ChemSusChem 2024, 17, e202400551 10.1002/cssc.202400551. 38618906

[ref45] Li J.; Ye Y.; Ye L.; Su F.; Ma Z.; Huang J.; Xie H.; Doronkin D. E.; Zimina A.; Grunwaldt J.-D.; Zhou Y. Sunlight induced photo-thermal synergistic catalytic CO_2_ conversion via localized surface plasmon resonance of MoO3-x. J. Mater. Chem. A 2019, 7, 2821–2830. 10.1039/C8TA10922B.

[ref46] Yang Y.; Shen Z.; Yang H.; Zou X.; Meng Y.; Jiang L.; Liu Y.; Xia Q.; Cao Y.; Li X.; Gao J.; Wang Y. Construction adsorption and photocatalytic interfaces between C, O co-doped BN and Pd-Cu alloy nanocrystals for effective conversion of CO_2_ to CO. J. Colloid Interface Sci. 2023, 640, 949–960. 10.1016/j.jcis.2023.02.146. 36907155

[ref47] Dong W.-W.; Jia J.; Wang Y.; An J.-R.; Yang O.-Y.; Gao X.-J.; Liu Y.-L.; Zhao J.; Li D.-S. Visible-light-driven solvent-free photocatalytic CO_2_ reduction to CO by Co-MOF/Cu_2_O heterojunction with superior selectivity. Chemical Engineering Journal 2022, 438, 135622 10.1016/j.cej.2022.135622.

[ref48] Khoshooei M. A.; Wang X.; Vitale G.; Formalik F.; Kirlikovali K. O.; Snurr R. Q.; Pereira-Almao P.; Farha O. K. An active, stable cubic molybdenum carbide catalyst for the high-temperature reverse water-gas shift reaction. Science 2024, 384, 540–546. 10.1126/science.adl1260. 38696554

[ref49] Atsbha T. A.; Yoon T.; Seongho P.; Lee C.-J. A review on the catalytic conversion of CO_2_ using H_2_ for synthesis of CO, methanol, and hydrocarbons. J. CO2 Util. 2021, 44, 101413 10.1016/j.jcou.2020.101413.

[ref50] Nirmal L. A.; Bhakthochidan S. A.; Vishal R.; Roshini V. B.; Jacob S. In Biofuels and Bioenergy; Gurunathan B., Sahadevan R., Eds.; Elsevier, 2022; pp 323–346.

[ref51] Frei M. S.; Mondelli C.; García-Muelas R.; Kley K. S.; Puértolas B.; López N.; Safonova O. V.; Stewart J. A.; Curulla Ferré D.; Pérez-Ramírez J. Atomic-scale engineering of indium oxide promotion by palladium for methanol production via CO_2_ hydrogenation. Nat. Commun. 2019, 10, 3377 10.1038/s41467-019-11349-9. 31358766 PMC6662860

[ref52] Verma N.; Delhez R.; van der Pers N. M.; Tichelaar F. D.; Böttger A. J. The role of the substrate on the mechanical and thermal stability of Pd thin films during hydrogen (de)sorption. Int. J. Hydrogen Energy 2021, 46, 4137–4153. 10.1016/j.ijhydene.2020.10.163.

[ref53] Galinski H.; Ryll T.; Schlagenhauf L.; Rechberger F.; Ying S.; Gauckler L. J.; Mornaghini F. C.; Ries Y.; Spolenak R.; Döbeli M. Dealloying of platinum-aluminum thin films: dynamics of pattern formation. Physical review letters 2011, 107, 225503 10.1103/PhysRevLett.107.225503. 22182033

[ref54] Alvarez-Garcia A.; Flórez E.; Moreno A.; Jimenez-Orozco C. CO_2_ activation on small Cu-Ni and Cu-Pd bimetallic clusters. Molecular Catalysis 2020, 484, 110733 10.1016/j.mcat.2019.110733.

