# Supplementary material for: CO2 Conversion in Cu–Pd Based Disordered Network Metamaterials with Ultrasmall Mode Volumes
Source: Nano Lett. 2025 Feb 20;25(10):3740–6. doi: 10.1021/acs.nanolett.4c05426 (PMC11907637; doi:10.1021/acs.nanolett.4c05426)
Supplement: Supplementary file 1 — nl4c05426_si_001.pdf [file nl4c05426_si_001.pdf]

Supporting Information

CO_2 Conversion in Cu-Pd based Disordered Network Metamaterials with Ultra-Small Mode Volumes

Jelena Wohlwend,*,† Oliver Wipf,† David Kiwic,‡ Siro Käch,† Benjamin Mächler,† Georg Haberfehlner,¶ Ralph Spolenak,† and Henning Galinski†

†*Laboratory for Nanometallurgy, Department of Materials, ETH Zurich, 8093 Zürich, Switzerland*

‡*Laboratory for Multifunctional Materials, Department of Materials, ETH Zurich, 8093 Zürich, Switzerland*

¶*Institut für Elektronenmikroskopie und Nanoanalytik, TU Graz, 8010 Graz; Austria*

E-mail: jelena.wohlwend@mat.ethz.ch

Analysis of Network Architecture

The network architecture of the Cu-Pd metamaterials is characterized by means of the networks mean strut length l_s, its mean pore intercept length L_m and the void fraction ϕ. Networks of four different compositions, namely Pd, $Cu_{.57}Pd_{.43}$, $Cu_{.75}Pd_{.25}$ and Cu were analyzed combining high resolution scanning electron microscopy (SEM) with standard image analysis using ImageJ.[1] SEM images in Figure S1A-D show the self-assembled plasmonic networks after chemical dealloying in a sodium hydroxide solution. Although all systems are interconnected, the addition of Cu induces a transition from a coral-like structure to a network of particles with a considerably higher void fraction (Fig.S1A to Fig.S1B). At higher

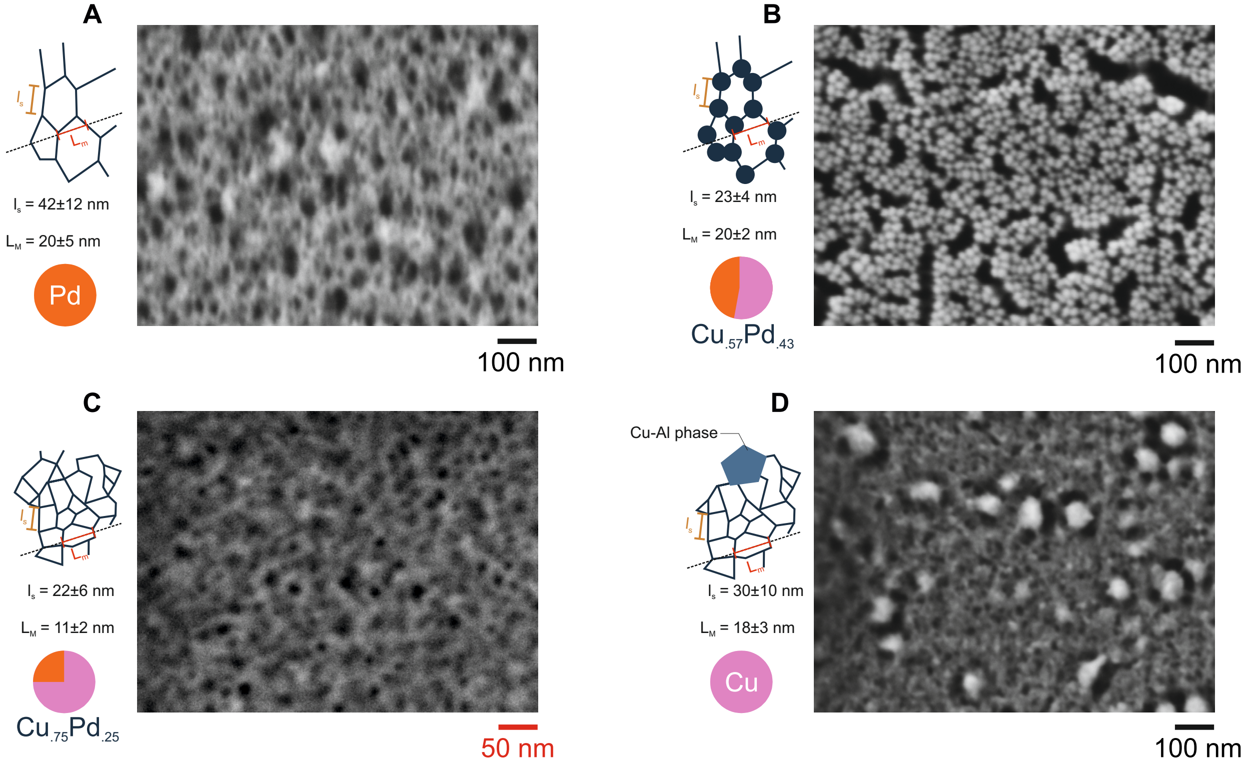

Figure S1: **High resolution top view SEM micrographs of plasmonic networks A** Self-assembled Pd-based network resulting from chemical dealloying. The network exhibits a disordered coral-like architecture with features sizes, such as mean strut length l_s and mean pore intercept length L_m, in the nanometer range. **B** $Cu_{.57}Pd_{.43}$ network exhibiting a network of particles with a considerably higher void fraction $\phi = 30\%$ **C** $Cu_{.75}Pd_{.25}$ ultra-fine plasmonic network with reduced l_s and L_m. We assume that due to increase of the specific surface area SSA, both the LDOS and catalytic activity of is increased. **D** Cu networks exhibiting as a disordered coral-like architecture with embedded nanoparticles, likely due to the formation of an Cu-Al intermetallic phase during chemical dealloying.

Cu content, i.e. $Cu_{.75}Pd_{.25}$, the network topology is reverted to a significantly refined coral-like structure (Fig.S1B to Fig.S1C). The strut length $l_s = 22 \pm 6$ and mean pore intercept length $L_m = 11 \pm 2$ are reduced, resulting in an increase of the specific surface area SSA and the LDOS (see main text). The "pure" Cu-networks exhibit as well a coral-like architecture but also shows the aggregation of a dispersed secondary phase, in form of nanoparticles.

According to EDX analysis the residual aluminum content is below the site percolation threshold of a three-dimensional fcc lattice $p_c = 0.198$[2] for all plasmonic networks except the "pure" copper networks. In the case of "pure" copper networks, the residual Al content is $c_{Al} = 0.32$, which is also reflected in the formation of nanoparticles in the network

Fig. S1D. Such formation might be due to the decomposition in two intermetallic phases during chemical dealloying.[3]

Surface Area and Weight Calculation of Cu-Pd disordered Network Metamaterials

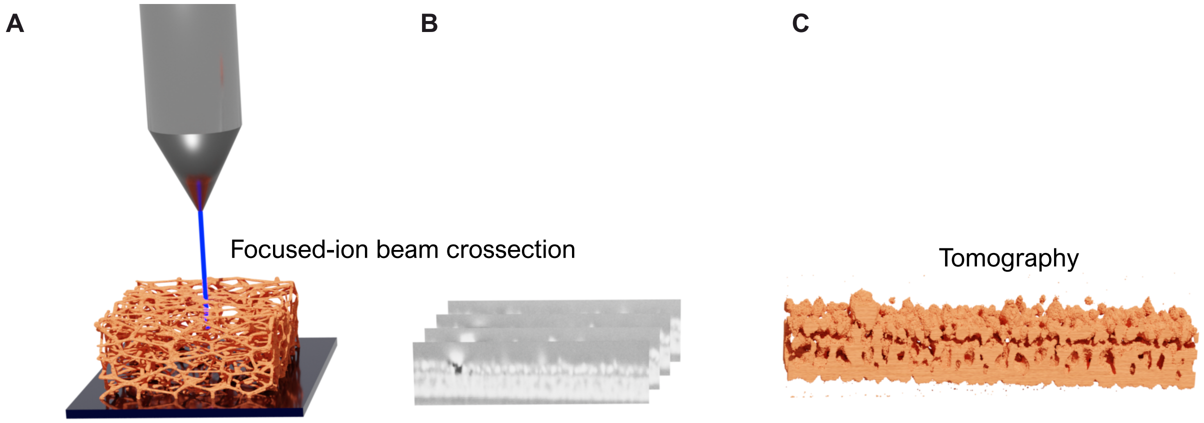

Figure S2: **Focused-ion-beam Tomography Workflow A** Schematic illustration of the focused-ion-beam crosssection cutting. **B** Scanning electron micrograph of several crosssection of a selected network. **C** Reconstructed 3D model showing the network architecture.

Focused ion beam (FIB) cross-sections were utilized to generate tomographic data, which were subsequently used to calculate both the surface area and mass of the catalyst using the open-source software Blender. These values were then employed to determine the catalytic yield of the disordered network metamaterial (DNM) in grams per hour. The schematic workflow is illustrated in Figure S2.

The process includes sequential FIB cross-sectioning, where each cut is followed by the acquisition of a scanning electron micrograph. These micrographs were binarized and compiled into a stack. Using Blender, a 3D reconstruction of the catalyst was created from the image stack. Using the Add-On "3D Print Toolbox[4]" the surface area and volume was measured on the three-dimensional tomographies. Using EDX measurements, the density of the networks was approximated by calculating an effective density based on the concentration

of the single components. Using the effective density and the network volume, the weight of the network g_{cat} has been calculated.

Figure S3 presents focused ion beam (FIB) tomography images taken before and after catalytic experiments. The images reveal pore coarsening and the formation of nanoparticles on the DNM surface. Please note that for calculating the production rates we used the surface area determined from tomographies post-catalysis.

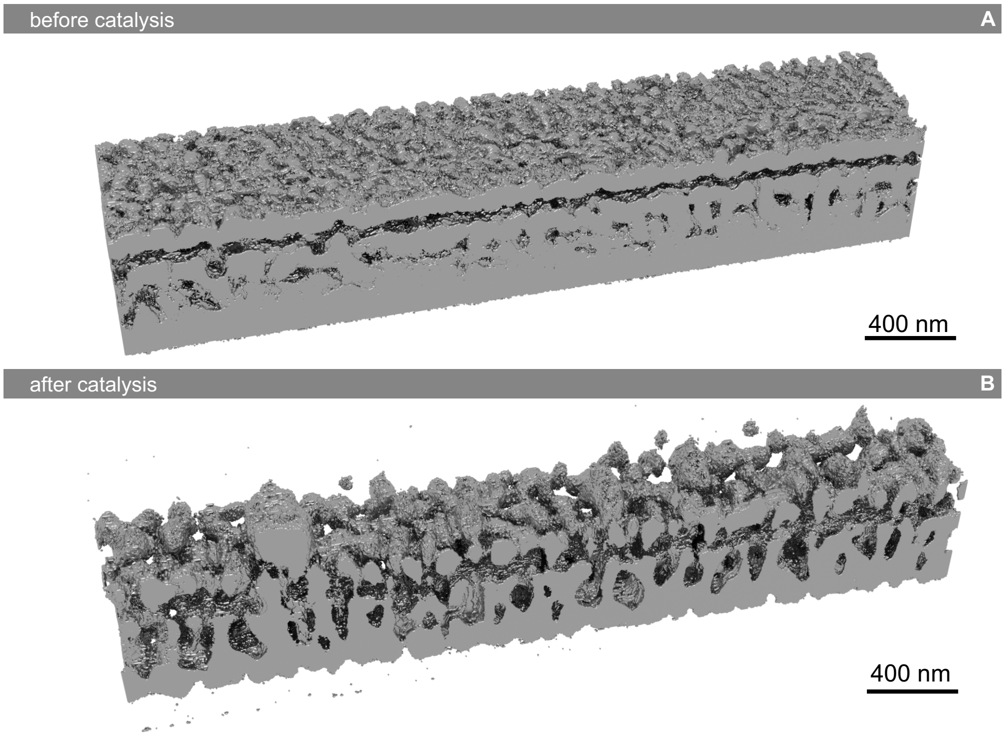

Figure S3: **Focused-ion-beam Tomography** Tomography Reconstructions of the Cu-Pd disordered network metamaterials from FIB Crosssections, before **A** and after **B** the catalysis experiments. These 3D models were used to determine the surface area and the volume which were then used to calculate the absolute catalysis yields per weight and surface area of the samples. The specific surface area SSA of each network composition is summarized in the main text (Table 1)

Activation Energy

From the temperature dependent yield shown in Figure 2 **C**, the activation energy can be derived, for the first reaction step, the conversion of CO_2 to CO and H_2O. This is done by deriving the reaction rate $r(T)$ at different temperatures (at 225°C, 250°C, 275°C and at 300°C).

$$r_{225^\circ} = \frac{d[CO]}{dt} = \frac{M \cdot c(CO) \cdot V_{gas}}{t} = \frac{0.044\,64\,\mathrm{mol\,L^{-1}} \cdot 0.000\,115\,\mathrm{L^{-1}} \cdot 0.01L}{60s} = 8.557 \cdot 10^{-10}\mathrm{mol/(L \cdot s)} \quad (1)$$

$$r_{300^\circ} = \frac{d[CO]}{dt} = \frac{M \cdot c(CO) \cdot V_{gas}}{t} = \frac{0.044\,64\,\mathrm{mol/L} \cdot 0.001\,308\,\mathrm{L^{-1}} \cdot 0.01L}{60s} = 9.732 \cdot 10^{-9}\mathrm{mol/(L \cdot s)} \quad (2)$$

Equations 1 and 2 show the calculation for 225 and 300 ° C. With r the reaction rate, $\frac{d[CO]}{dt}$ the carbon monoxide change, M the moles of molecules per liter of gas, $c(CO)$ the percentage of carbon monoxide molecules per liter of gas, V_{gas} the amount of gas flowing through the reactor per minute and t the number of seconds per minute.

In a next step this rate can be converted into the corresponding reaction rate constant $k(T)$.

$$k_{225^\circ} = \frac{r_{225^\circ}}{[H_2] \cdot [CO_2]} = \frac{8.557 \cdot 10^{-10}\mathrm{mol/(L \cdot s)}}{0.9 \cdot 0.04464 \cdot 0.1 \cdot 0.04464} = 4.770 \cdot 10^{-6}\mathrm{L/(mol \cdot s)} \quad (3)$$

$$k_{300^\circ} = \frac{r_{300^\circ}}{[H_2] \cdot [CO_2]} = \frac{4.323 \cdot 10^{-9}\mathrm{mol/(L \cdot s)}}{0.9 \cdot 0.04464 \cdot 0.1 \cdot 0.04464} = 5.426 \cdot 10^{-5}\mathrm{L/(mol \cdot s)} \quad (4)$$

With k being the reaction rate constant, $[H_2]$ the hydrogen concentration per liter and

$[CO_2]$ the carbon dioxide concentration per liter before the reaction. Equation 3 and Equation 4 thereby show the conversion from the reaction rate into the reaction rate constant $k(T)$.

We can now represent this data in an Arrhenius plot (see Figure 2 **D**), where the natural logarithm of the reaction rate constant is plotted against the inverse temperature. The slope, m (Equation 6), of the linear fit to the temperature dependence of k(T), corresponds to the activation energy for the reduction of CO_2 to CO (Equation 7).

The equation of the linear fit is:

$$y = -9770.2x + 1.63 \tag{5}$$

allowing to calculate the slope m:

$$m = -9770.2\,\mathrm{K}^{-1} \tag{6}$$

Then, from the slope m, the activation Energy E_a is calculated as followed:

$$E_a = -m \cdot R = 9770.2\,\mathrm{K}^{-1} \cdot 8.314\,\mathrm{J/mol} = 81.05\,\mathrm{kJ/mol} = 0.84(10)\,\mathrm{eV/molecule} \tag{7}$$

The calculated activation energy for $\mathrm{CO_2}$ reduction is 81.05 kJ/mol or 0.84(10) eV per molecule. The calculated activation energy is roughly comparable and in the same order of magnitude with findings by Frei et al.[5]

Plasmonic and Catalytic Metals

Plasmonic metals have their Fermi energy well above the d-band (Figure S4 **A**). This results in intra-band plasmons (from the s- to s-band) upon light illumination, until the energy required for inter-band (d- to s-band) transitions is reached. The energies of electromagnetic

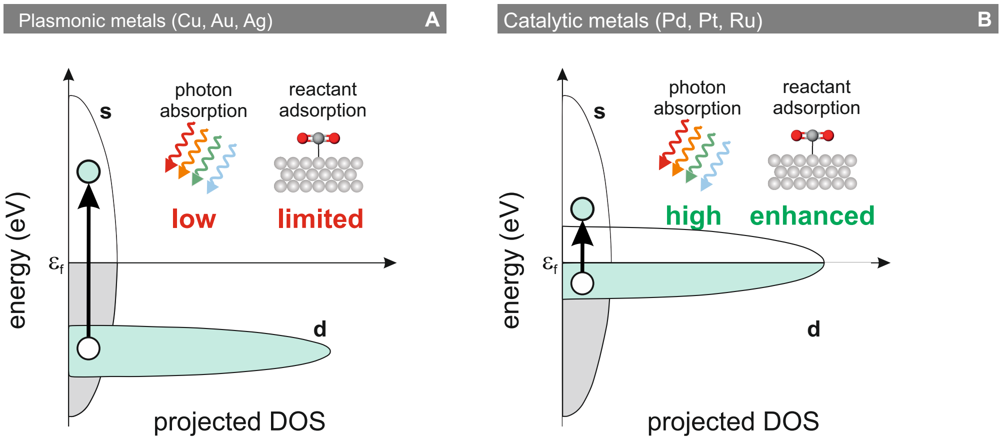

Figure S4: **Projected DOS of plasmonic and catalytic metals. A** The electronic band structure of plasmonic metals, i.e. noble metals, such as Cu, Au, and Ag. These metals exhibit a completely filled d-band situated well below the Fermi level ε_f. Only high energy photons outside the visible range can be absorbed via interband excitations (d-s). Here, the d-band centers are low in energy relative to the Fermi level, which inhibits reactant adsorption and low catalytic activity or high nobility. **B** Electronic band structure of catalytic metals, i.e. transition metals, such as Pd, Pt and Ru. These metals exhibit a not completely filled d-band which cross the Fermi level ε_f. Photons absorption in visible range is enhanced via interband excitations (d-s) and intraband excitations, see also Ref.[6] Here, the d-band centers are high in energy relative to the Fermi level, which enhances reactant adsorption and increases the catalytic activity of the material. Figure adapted from Ref.[7]

waves that generate surface plasmons fall within the visible light spectrum, making metals with this electronic structure highly effective plasmonic materials that absorb visible light. Examples of such metals include copper, gold, and silver.[8]

In contrast, metals used in catalysis have their Fermi energy intersecting the d-band (Figure S4 **B**), which leads to an optical response dominated by inter-band transitions. These inter-band transitions dampen localized surface plasmon resonance, resulting in reduced optical absorption. Additionally, catalytic metals are mostly optically responsive in the ultraviolet (UV) range, often requiring precise geometric engineering to shift the response into the visible light spectrum.[9]

Since the s- and p-bands of transition metals are relatively uniform across elements, their ability to interact with molecules depends on the d-band. The narrow d-band hybridizes molecular orbitals into bonding and anti-bonding states, with only the bonding states lying

below the Fermi energy, corresponding to the chemisorption of molecules.[10] Examples of such catalytic metals include platinum, ruthenium, titanium, and palladium (Figure S4 **B**). By combining plasmonic metals with catalytic metals, bimetallic nanostructures can be created that simultaneously enhance light conversion and promote strong molecular adsorption.[11]

Catalytic performance

Figure S5, S6 and S7 report the catalytic conversion data obtained at $T = 300°C$ over monometallic Pd and Cu networks and a bimetallic $Cu_{.53}Pd_{.47}$ network.

Figure S8 shows the yield for two different networks, both with and without illumination. DNMs containing Cu exhibit an increase in production and a shift in selectivity with illumination, while DNMs without Cu (composed solely of Pd) do not show any increase in yield or change in selectivity. The detailed evolution of the selectictivity as function of light condition (dark, white) is summarized in Table S1.

Figure S9 shows representative gas chromatography (GC) spectra confirming the detection of only CO, CH_4, C_2H_6 and C_2H_4 as products that exceed 0.1mmol $g^{-1}h^{-1}$.

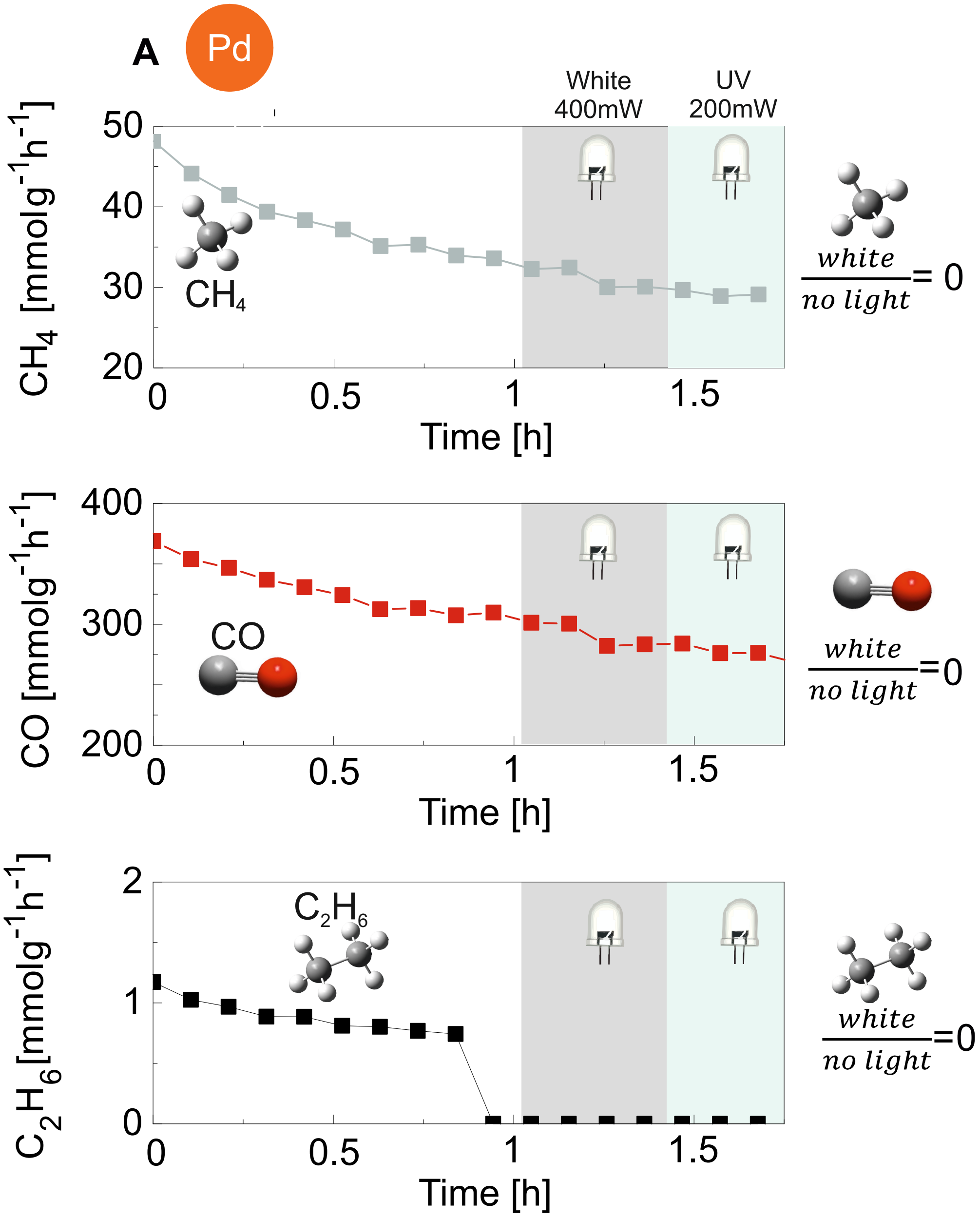

Figure S5: "Light-assisted" CO_2 conversion at constant temperature ($T_{max} = 300°C$) in a Pd-network as a function of time. The Pd network promotes the reduction of CO_2 to CO with high selectivity $S_{CO} = 90\%$. The conversion enhancement with white LED illumination (vs. no light) is depicted on the right. No enhancement or change in selectivity due to white LED illumination is detected. A decrease in production as function of time is observed in all reaction products indicating degradation of the catalyst.

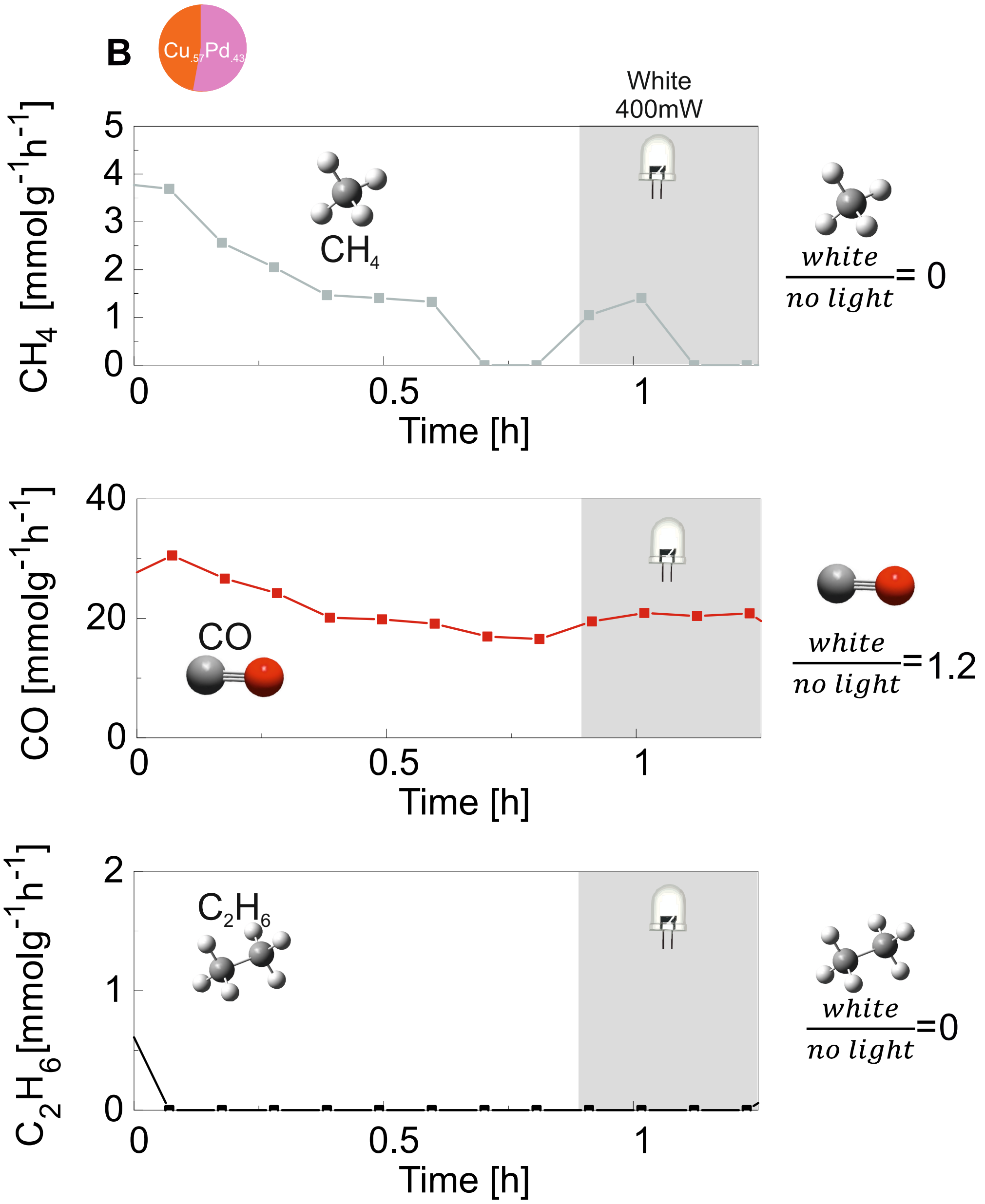

Figure S6: "Light-assisted" CO_2 conversion at constant temperature ($T_{max} = 300°C$) in a $Cu_{.53}Pd_{.47}$-network as a function of time. The $Cu_{.53}Pd_{.47}$ network promotes the reduction of CO_2 to CO with high selectivity $S_{CO} = 100\%$ at a overall low activity. The conversion enhancement with white LED illumination (vs. no light) is depicted on the right. For CO and C_2H_6 no enhancement or change in selectivity due to white LED illumination is detected. The CH_4 production rate is slightly enhanced under illumination.

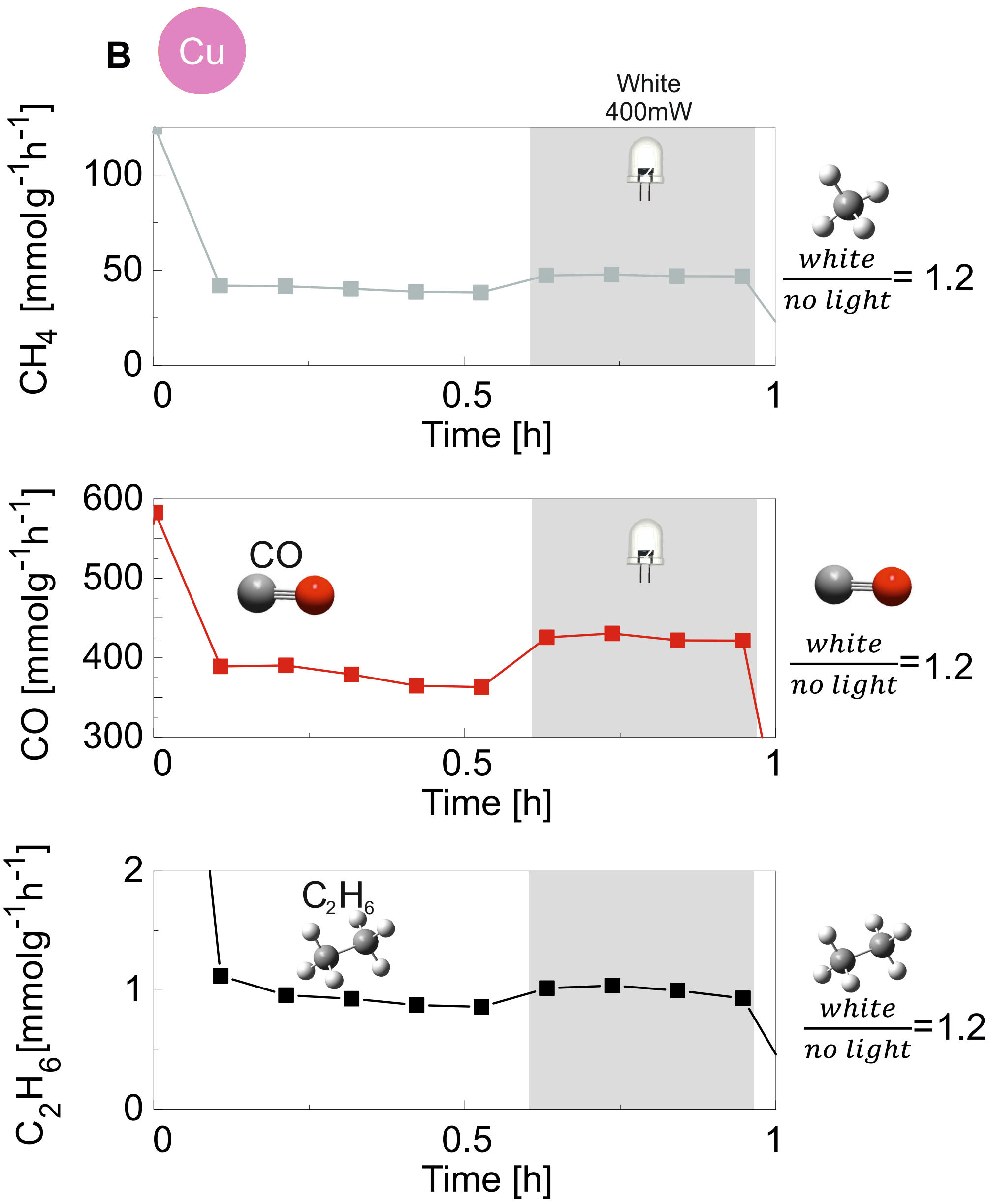

Figure S7: "Light-assisted" CO2 conversion at constant temperature (T_{max} = 300°C) in a Cu-network as a function of time. The Cu network promotes the reduction of CO_2 to CO with high selectivity S_{CO} = 90.3%. The conversion enhancement with white LED illumination (vs. no light) is depicted on the right. The production rate for all products is enhanced equally under white LED illumination.

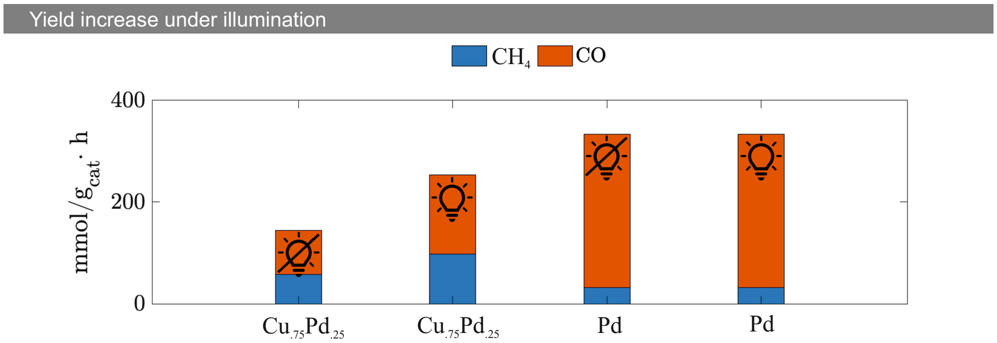

Figure S8: **Comparison of yield with and without illumination for $Cu_{.75}Pd_{.25}$ and Pd.** While the presence of light has no measurable influence on the yield of the Pd network, the yield increases under illumination in the $Cu_{.75}Pd_{.25}$ and Pd.

Table S1: Measured selectivity of different DNMs.

	S_{CH_4} (%)		S_{CO} (%)		$S_{C_2H_6}$ (%)		$S_{C_2H_4}$ (%)		$S_{C_2H_2}$ (%)	
Phase	L_{off}	L_{on}	L_{off}	L_{on}	L_{off}	L_{on}	L_{off}	L_{on}	L_{off}	L_{on}
Cu	9.5	9.9	90.3	89.8	0.2	0.2	0	0	0	0
$Cu_{.75}Pd_{.25}$	44.2	39.0	55.2	50.4	0.6	0.6	0	0.2	0	0
$Cu_{.53}Pd_{.47}$	0	6.3	100	93.7	0	0	0	0	0	0
Pd	9.7	9.7	90.3	90.3	0	0	0	0	0	0

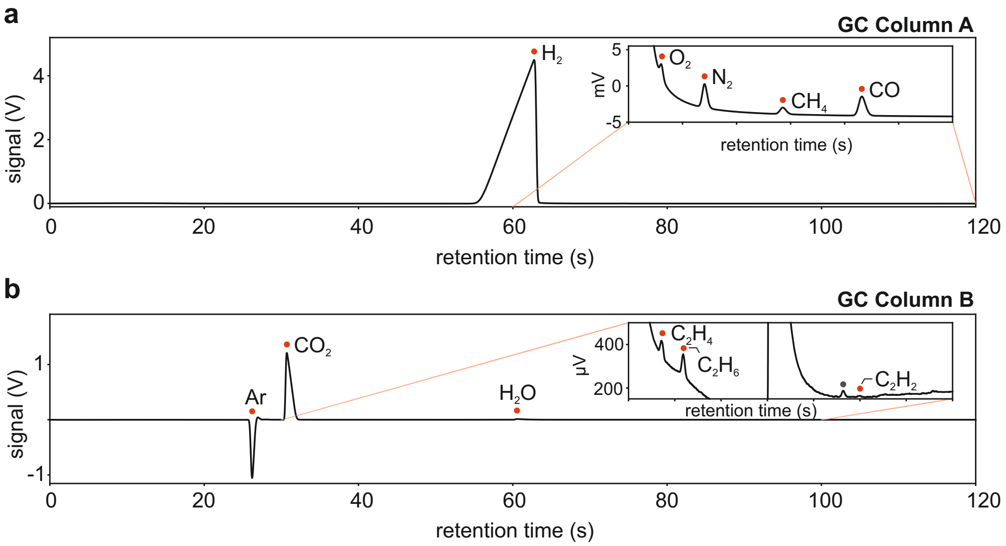

Figure S9: **Gas chromatography spectra for a $Cu_{.75}Pd_{.25}$ network.** The gas chromatography spectra show peaks for the detected molecules synthesized during the photocatalytic conversion of CO_2 and H_2 in two GC columns (A,B). (a) GC column A is calibrated to detect H_2, O_2, N_2, CH_4 and CO. The spectrum shows expected compounds from the catalytic conversion of CO_2 and H_2. Oxygen and nitrogen peaks result from residual O_2 and N_2 in the gas line. (b) GC column B is calibrated to detect Ar, CO_2, H_2O and hyrdocarbons such as C_2H_4, C_2H_6 and C_2H_2. Argon is used as carrier gas in the GC columns. One unidentified peak is present, highlighted by the grey dot, that can not be identified with the used calibration setting.

References

(1) Schneider, C. A.; Rasband, W. S.; Eliceiri, K. W. NIH Image to ImageJ: 25 years of image analysis. *Nature Methods* **2012**, *9*, 671–675.

(2) Stauffer, D.; Aharony, A. In *Encyclopedia of Physical Science and Technology (Third Edition)*, third edition ed.; Meyers, R. A., Ed.; Academic Press: New York, 2003; pp 655–669.

(3) Wohlwend, J.; Haberfehlner, G.; Galinski, H. Strong Coupling in Two-Phase Metamaterials Fabricated by Sequential Self-Assembly. *Advanced Optical Materials* **2023**, *11*, 2300568.

(4) Pas, A. 2019; `https://github.com/agapas/3d-print-toolbox-modified`.

(5) Frei, M. S.; Mondelli, C.; García-Muelas, R.; Kley, K. S.; Puértolas, B.; López, N.; Safonova, O. V.; Stewart, J. A.; Curulla Ferré, D.; Pérez-Ramírez, J. Atomic-scale engineering of indium oxide promotion by palladium for methanol production via CO2 hydrogenation. *Nature communications* **2019**, *10*, 3377.

(6) Lyu, P.; Espinoza, R.; Nguyen, S. C. Photocatalysis of Metallic Nanoparticles: Interband vs Intraband Induced Mechanisms. *The Journal of Physical Chemistry C* **2023**, *127*, 15685–15698.

(7) Nilsson, A.; Pettersson, L.; Hammer, B.; Bligaard, T.; Christensen, C. H.; Nørskov, J. K. The electronic structure effect in heterogeneous catalysis. *Catalysis letters* **2005**, *100*, 111–114.

(8) Wang, L.; Hasanzadeh Kafshgari, M.; Meunier, M. Optical Properties and Applications of Plasmonic-Metal Nanoparticles. *30*.

(9) Langhammer, C.; Yuan, Z.; Zorić, I.; Kasemo, B. Plasmonic Properties of Supported Pt and Pd Nanostructures. *6*, 833–838.

(10) Hammer, B.; Nørskov, J. *Impact of Surface Science on Catalysis*; Elsevier, pp 71–129.

(11) Sytwu, K.; Vadai, M.; Dionne, J. A. Bimetallic nanostructures: combining plasmonic and catalytic metals for photocatalysis. *4*, 1619480.
